# A systematic review and quality assessment of complementary and alternative medicine recommendations in insomnia clinical practice guidelines

**DOI:** 10.1186/s12906-021-03223-3

**Published:** 2021-02-08

**Authors:** Jeremy Y. Ng, Nandana D. Parakh

**Affiliations:** Department of Health Research Methods, Evidence, and Impact, Faculty of Health Sciences, McMaster University, Michael G. DeGroote Centre for Learning and Discovery, Room 2112, 1280 Main Street West, Hamilton, ON L8S 4K1 Canada

**Keywords:** AGREE II, Clinical practice guideline, Complementary and alternative medicine, Insomnia, Sleep disorders, Systematic review

## Abstract

**Background:**

Sleep disorders encompass a wide range of conditions which affect the quality and quantity of sleep, with insomnia being a specific type of sleep disorder of focus in this review. Complementary and alternative medicine (CAM) is often utilized for various sleep disorders. Approximately 4.5% of individuals diagnosed with insomnia in the United States have used a CAM therapy to treat their condition. This systematic review identifies the quantity and assesses the quality of clinical practice guidelines (CPGs) which contain CAM recommendations for insomnia.

**Methods:**

MEDLINE, EMBASE and CINAHL were systematically searched from 2009 to 2020, along with the Guidelines International Network, the National Center for Complementary and Integrative Health website, the National Institute for Health and Care Excellence, and the Emergency Care Research Institute. CPGs which focused on the treatment and/or management of insomnia in adults were assessed with the Appraisal of Guidelines, Research and Evaluation II (AGREE II) instrument.

**Results:**

From 277 total results, 250 results were unique, 9 CPGs mentioned CAM for insomnia, and 6 out of the 9 made CAM recommendations relevant to insomnia. Scaled domain percentages from highest to lowest were scope and purpose, clarity of presentation, editorial independence, stakeholder involvement, rigour of development, and applicability. Quality varied within and across CPGs.

**Conclusions:**

The CPGs which contained CAM recommendations for insomnia and which scored well could be used by health care professionals and patients to discuss the use of CAM therapies for the treatment/management of insomnia, while CPGs which scored lower could be improved in future updates according to AGREE II.

**Supplementary Information:**

The online version contains supplementary material available at 10.1186/s12906-021-03223-3.

## Background

Sleep disorders are classified as a broad class of disorders which center around changes in quality and quantity of sleep, and can affect a patient's safety, productiveness, and overall quality of life [1, 2]. Sleep disorders include conditions such as insomnia, obstructive sleep apnea, narcolepsy, and restless leg syndrome [1, 2]. Insomnia is one of the most common sleep disorders and is defined as difficulty in initiating sleep, maintaining sleep, or obtaining good quality sleep [1–4]. Insomnia has been linked with many comorbid disorders such as hypertension, cardiovascular disease, depression, and diabetes [5]. Insomnia can also result in alterations to attention and episodic memory, and these cognitive impairments are clinically significant [6]. Thus, the treatment of insomnia is important for managing the overall health of an individual. Insomnia can be diagnosed as acute or chronic; furthermore, primary insomnia is defined as an individual developing sleep-preventing associations due to stress or emotions, while co-morbid insomnia is sleeplessness which arises from a primary illness [2, 7]. Specific diagnostic criteria for insomnia can be found in the International Classification of Sleep Disorders guideline [8]. Approximately 70 million Americans suffer from a sleep disorder in general, and more specifically, a 2018 study found that approximately 25% of the adult population experiences acute insomnia in the United States [9, 10]. One common group of therapies used by those suffering from insomnia include complementary and alternative medicine (CAM). Among adults experiencing insomnia, research has shown that 4.5% of them utilized CAM to treat their condition [11]. “Complementary medicine” is defined as practices which are considered to be non-mainstream, or methods which are not a part of the standard medical care. They are used *in conjunction* with conventional medical practices [12, 13]. In contrast, “alternative medicine” is defined as a non-mainstream practice which is used *instead* of standard medical care [12, 13].

CAM consists of a wide range of therapies which originate from various schools of thought as well as geographical regions of the world [14, 15]. Common CAM therapies used for insomnia include acupuncture, Ayurveda, melatonin, valerian, yoga, and mind-body practices [15–21]. Mind-body interventions such as mindfulness meditation and yoga assist in stress management/reduction and management of anxiety, which can improve sleep quality [17]. Melatonin, a highly studied CAM treatment for insomnia, targets the pineal gland and influences the circadian sleep-wake cycle, along with having possible sedative effects [18, 19]. Acupuncture and acupressure have been proposed to restore the normal sleep-wake cycle, and can also be used to increase the content of γ-amino butyric acid which enhances quality of sleep [20, 21]. Acupuncture has also been used to manage fatigue [20, 21]. While some individuals who suffer from insomnia utilize CAM, conventional healthcare providers generally receive little to no education or training about CAM therapies [22]. This lack of training can lead to miscommunication between healthcare providers and patients when treating insomnia with CAM, which can hinder the efficacy of the treatment plan [23].

Healthcare providers use evidence-based clinical practice guidelines (CPGs) to inform their practice decisions, especially in fields where their knowledge and expertise may be lacking [24]. Developers of CPGs create recommendations by reviewing current evidence, and assessing the benefits and risks associated with certain therapies [25]. The purpose of this present study was to conduct a systematic review to identify the quantity and assess the quality of CAM recommendations in CPGs for the treatment and/or management of insomnia using the AGREE II instrument.

## Methods

### Approach

In order to identify eligible CPGs focused on insomnia or containing a section on insomnia, standard methods and Preferred Reporting Items for Systematic Reviews and Meta-Analyses (PRISMA) criteria were utilized [26]. A protocol was registered with PROSPERO, registration number CRD42020182236. Eligible CPGs containing CAM recommendations for insomnia were assessed with the Appraisal of Guidelines, Research and Evaluation II (AGREE II) instrument, a tool which has been validated and widely-used [27]. CPGs which contained CAM recommendations were re-assessed with the AGREE II instrument and the assessors applied the 23 criteria to only the CAM sections of the CPGs. The AGREE II instrument contains 23 items grouped in 6 domains: scope and purpose, stakeholder involvement, rigor of development, clarity and presentation, applicability, and editorial independence.

### Eligibility criteria

Eligibility criteria for insomnia CPGs were based on the Population, Intervention, Comparison and Outcomes (PICO) framework. *Populations* which were considered eligible included adults aged 19 years and older with insomnia. With regards to *interventions*, we only included CPGs which 1) exclusively focused on the treatment and/or management of insomnia, or 2) focused on sleep disorders in general, but provided treatment and/or management recommendations for insomnia. CPGs which did not contain any mention of insomnia or CPGs which only provided recommendations for other sleep disorders (i.e. sleep apnea, restless leg syndrome) were excluded. *Comparisons* concerned the assessed quality of insomnia CPGs*.* The *outcomes* were scores from the AGREE II instrument which reflected CPG content and format. Additional inclusion criteria were applied to each CPG as follows: developed by non-profit organizations (this included academic institutions, government agencies, disease-specific foundations, or professional associations or societies); published in 2009 or later; published in the English language; and either publicly available or orderable via our library system. Publications which were deemed ineligible included protocols, abstracts, conference proceedings, letters and editorials. Additionally, primary studies (i.e., surveys, trials, case-control and cohort studies that evaluated an outcome relating to the treatment/management of insomnia, or focused on insomnia curriculum, education, training, research, professional certification or performance were all not eligible. The AGREE II instrument was applied twice to the eligible CPGs containing CAM recommendations: once for the overall CPGs, and then once for the CAM sections of the CPGs. This identified the domain score differences between the overall CPG and the CAM sections of the CPG. For eligible CPGs which only made mention of CAM, but did not contain CAM therapy recommendations, only demographic information was reported.

### Searching and screening

MEDLINE, EMBASE and CINAHL were searched on April 17, 2020 from 2009 to April 16, 2020 inclusive. The search strategy (Supplementary File 1) included indexed headings and keywords that reflect terms commonly used in the literature to refer to insomnia. We also searched the Guidelines International Network, a repository of guidelines by using keyword searches based on the eligibility criteria, such as “insomnia” and “sleep” [28]. We then searched the NCCIH website which contained a single list of CAM CPGs [29]. The National Institute for Health and Care Excellence (NICE) Evidence Search was searched using the terms “sleep” and “sleep conditions” [30], and the Emergency Care Research Institute (ECRI) Guidelines Trust was searched using the search term “insomnia” and specifying the type of publication as “guidance” [31]. NDP and another research assistant screened titles and abstracts from all sources. Then, NDP and the other research assistant screened full-text items to confirm eligibility as per the PICO guidelines. JYN reviewed the screened titles and abstracts and full-text items to standardize screening, and assisted in resolving discrepancies between the 2 screeners.

### Data extraction and analysis

Relevant data were collected from each CPG and summarised as follows: date of publication, country of first author, type of CPG publishing entity (i.e. research institutions, government departments, disease-specific foundations or professional associations or societies) and whether the specific CPG made any mention of or recommended any CAMs. Data extraction was performed on the type(s) of CAM listed, CAM recommendations made, CAM funding sources, and whether any CAM providers were part of the guideline panel in the event of CAM being mentioned in the CPG. Most of the aforementioned data was available in the CPG; in order to provide additional information, each developer’s website was searched for associated knowledge-based tools to support the implementation of the recommendations.

### Guideline quality assessment

NDP and another research assistant completed all data extractions and AGREE II quality assessments of eligible CPGs. The initial step included NDP and the other research assistant participating in a pilot test of the AGREE II instrument by applying it to 3 separate CPGs, and discrepancies in scores were discussed with JYN and resolved. NDP and the other research assistant then proceeded to independently assess all eligible CPGs containing CAM therapy recommendations twice (i.e., once for the overall CPG, and once for only the CAM sections of the CPG). Six domains which contain 23 separate items were rated using a seven-point Likert scale from strongly disagree (1) to strongly agree (7) that the CPG contained the relevant criteria. The overall quality of each CPG (1 to 7) was also rated based on individual score items, and that information was used to recommend, recommend with modifications, or not recommend the CPG for use. The modified AGREE II questions used to score the CAM sections of each CPG are found in Supplementary File 2. NDP and the other research assistant then met with JYN and discussed and resolved discrepancies between the two appraisers' scores. For each CPG, each assessor's average of all 23 scored items was calculated separately and those two values were then combined to produce each CPG’s average assessment score. For each CPG, the total evaluation scores of both appraisers were combined to produce the average overall assessments. For each domain, scaled percentages were determined to allow for comparisons between the various aspects of each CPG. For each of the six domains, the scaled domain percentage was determined by taking the total of the two appraisers' individual scores within the domain, scaling it to the minimum and maximum possible score for that domain, and then translating it into a percentage. The average of the CPG’s scaled domain percentages was also created for each domain.

## Results

### Search results (Fig. 1)

Searches retrieved 277 items, 250 were unique, and 237 titles and abstracts were eliminated, leaving 13 full-text CPGs that were considered. Of those, 2 were not eligible because they did not contain a focus on insomnia (*n* = 2), 1 was not eligible primarily because it was not focused on CAM (*n* = 1), and 1 was focused on the evaluation/diagnosis of insomnia rather than the management/treatment of insomnia (*n* = 1), leaving 9 CPGs eligible for review [32–40]. Six out of the 9 CPGs made CAM therapy recommendations [32, 33, 35, 37–39]; of the remaining 3 CPGs, 1 CPG only contained mention of CAM [34], and 2 CPGs contained neither CAM mentions nor CAM recommendations [36, 40].

**Fig. 1:**
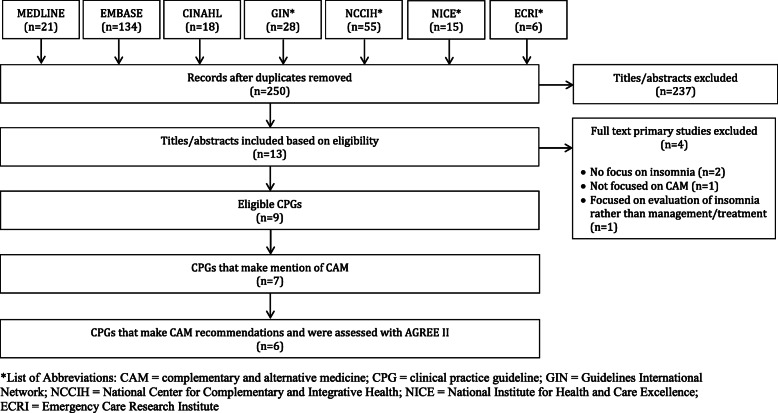
PRISMA Diagram

### Guideline characteristics (Table 1)

**Fig. 2:**
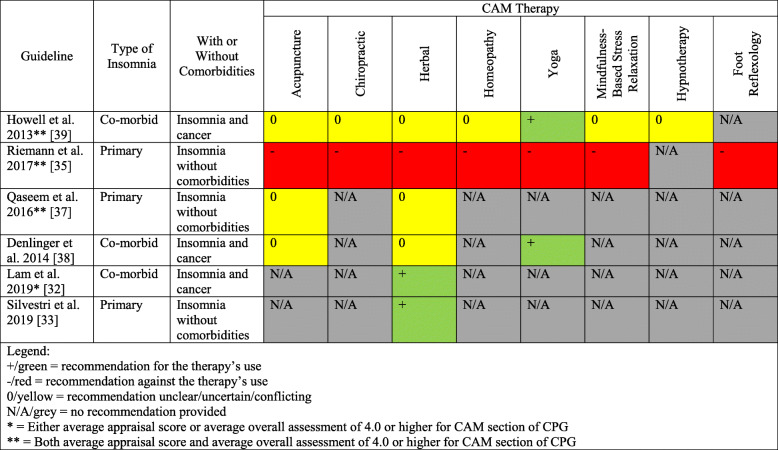
Summary of CAM Recommendations in Clinical Practice Guidelines

Eligible CPGs were published from 2009 to 2020 in the United States (*n* = 3), Brazil (*n* = 1), Canada (*n* = 1), Germany (*n* = 1), Hong Kong (*n* = 1), India (*n* = 1), and Italy (*n* = 1). The CPGs were funded and/or developed by professional associations or societies (*n* = 7), academic (*n* = 1), and an international agency (*n* = 1). Seven CPGs made mention of CAMs. CAM recommendations were made in 6 CPGs, and included herbal medicines (*n* = 4), acupuncture (*n* = 4), yoga (*n* = 3), chiropractic use (*n* = 2), homeopathy (*n* = 2), mindfulness-based stress relaxation (*n* = 2), physical activity (*n* = 2), hypnotherapy (*n* = 1), and foot reflexology (*n* = 1). Only the CPGs with CAM recommendations were assessed using the AGREE II tool. CAM funding sources were not used in any of the CPGs, and 1 CPG included CAM providers as part of the guideline panel. In Fig. 2, a summary of CAM recommendations for insomnia are provided for the benefit of clinicians and researchers.

**Table 1:** Characteristics of eligible guidelines

Guideline	Country (First Author)	Developer	CAM Category	Guideline Topic
Lam 2019 [32]	Hong Kong	Hong Kong Baptist University	Herbal treatments and Chinese medicine	Chinese medicine for cancer palliative care: pain, constipation, and insomnia
Silvestri 2019 [33]	Italy	Italian Association of Sleep Medicine	Melatonin	Pharmacologic & nonpharmacologic treatments for menopausal sleep disorders
Gupta 2017 [34]	India	Dept. of Psychiatry and Sleep Medicine, Himalayan Institute of Medical Science	Herbal, hypnotics, and mindfulness based stress relaxation	Pharmacologic & nonpharmacologic treatments for sleep disorders
Riemann 2017 [35]	Germany	European Sleep Research Society	Herbal medicine, acupuncture, yoga, chiropractic, homeopathy, mindfulness-based stress relaxation	Diagnosis & treatment of insomnia
Sateia 2017 [36]	United States	American Academy of Sleep Medicine	None	Pharmacologic treatment of insomnia
Qaseem 2016 [37]	United States	American College of Physicians	Acupuncture; Chinese herbal medicine	Pharmacologic & nonpharmacologic management & treatment of insomnia
Denlinger 2014 [38]	United States	National Comprehensive Cancer Network	Physical activity/yoga	Treatment & management of sleep disorders
Howell 2013 [39]	Canada	Cancer Journey Advisory Group of the Canadian Partnership Against Cancer	Herbal medicine, acupuncture, yoga, hypnotherapy, chiropractic, homeopathy, mindfulness-based stress relaxation	Treatment of sleep disturbances in cancer patients
Pinto 2010 [40]	Brazil	The Brazilian Sleep Association	None	Diagnosis and treatment of insomnia

### Guidelines mentioning CAM without recommendations

One CPG contained CAM mention but did not make specific CAM recommendations [34]. This CPG mentioned mindfulness-based stress relaxation, hypnotic agents, and various herbal therapies [34].

### Average appraisal scores, average overall assessments and recommendations regarding use of CPGs: overall CPG (Table 2)

On the seven-point Likert scale, the average appraisal scores for each of the 6 CPGs ranged from 3.8 to 5.9 (where 7 equals strongly agree that the item is met). All 6 CPGs achieved or exceeded a 3.0 average appraisal score, 4 CPGs achieved or exceeded a 4.0 average appraisal score, and 2 CPGs achieved or exceeded a 5.0 average appraisal score. The average overall assessment for the 6 CPGs ranged from 3.5 (lowest) to 6.0 (highest), which included 4 CPGs equal to or greater than 4.0 and 2 CPGs equal to or greater than 5.0.

**Table 2:** Average appraisal scores and average overall assessments of each guideline

Guideline	Metric	Appraiser 1	Appraiser 2	Average	Standard Deviation
Lam 2019 [32] (Overall)	Appraisal Score	3.8	4.5	4.2	0.7
	Overall Assessment	3.0	4.0	3.5	1.0
Lam 2019 [32] (CAM Section)	Appraisal Score	3.8	4.5	4.2	0.7
	Overall Assessment	3.0	4.0	3.5	1.0
Silvestri 2019 [33] (Overall)	Appraisal Score	3.8	3.8	3.8	0.0
	Overall Assessment	4.0	4.0	4.0	0.0
Silvestri 2019 [33] (CAM)	Appraisal Score	3.6	3.3	3.4	0.3
	Overall Assessment	3.0	3.0	3.0	0.0
Riemann 2017 [35] (Overall)	Appraisal Score	5.0	4.7	4.8	0.3
	Overall Assessment	6.0	5.0	5.5	1.0
Riemann 2017 [35] (CAM Section)	Appraisal Score	4.6	4.7	4.6	0.1
	Overall Assessment	5.0	5.0	5.0	0.0
Qaseem 2016 [37] (Overall)	Appraisal Score	5.8	6.0	5.9	0.2
	Overall Assessment	6.0	6.0	6.0	0.0
Qaseem 2016 [37] (CAM Section)	Appraisal Score	4.7	4.9	4.8	0.2
	Overall Assessment	6.0	6.0	6.0	0.0
Denlinger 2014 [38] (Overall)	Appraisal Score	3.5	3.5	3.5	0.0
	Overall Assessment	3.0	4.0	3.5	0.0
Denlinger 2014 [38] (CAM Section)	Appraisal Score	3.3	2.7	3.0	0.6
	Overall Assessment	2.0	3.0	2.5	1.0
Howell 2013 [39] (Overall)	Appraisal Score	6.0	5.7	5.8	0.3
	Overall Assessment	6.0	5.0	5.5	1.0
Howell 2013 [39] (CAM Section)	Appraisal Score	4.8	4.7	4.7	0.1
	Overall Assessment	5.0	4.0	4.5	1.0

### Average appraisal scores, average overall assessments and recommendations regarding use of CPGs: CAM sections (Table 2)

On the seven-point Likert scale, the average appraisal scores for each of the 6 CPGs ranged from 3.0 to 4.8 (where 7 equals strongly agree that the item is met). All 6 CPGs achieved or exceeded a 3.0 average appraisal score, 4 CPGs achieved or exceeded a 4.0 average appraisal score. The average overall assessment for the 6 CPGs ranged from 2.5 (lowest) to 6.0 (highest), which included 5 CPGs equal to or greater than 3.0 and 3 CPGs equal to or greater than 4.0.

### Overall recommendations: overall guideline (Table 3)

For overall recommendations, appraisers agreed in their overall recommendation for 3 out of 6 CPGs including Yes [39], Yes with modifications [33], and No [38]. Of the remaining 3 CPGs, 2 were rated Yes with modifications and Yes [35, 37], while the remaining CPG was rated No and Yes with Modifications [32].

**Table 3:** Overall recommendations for use of appraised guidelines

Guideline	Overall Guideline		CAM Section	
	Appraiser 1	Appraiser 2	Appraiser 1	Appraiser 2
Lam 2019 [32]	No	Yes with Modifications	No	Yes with Modifications
Silvestri 2019 [33]	Yes with Modifications	Yes with Modifications	No	No
Riemann 2017 [35]	Yes with Modifications	Yes	Yes with Modifications	Yes
Qaseem 2016 [37]	Yes with Modifications	Yes	Yes with Modifications	Yes
Denlinger 2014 [38]	No	No	No	No
Howell 2013 [39]	Yes	Yes	Yes with Modifications	Yes with Modifications

### Overall recommendations: CAM sections (Table 3)

For the CAM sections of the CPGs, 3 out of 6 CPGs were given the same appraisal, with 1 CPG being Yes [39], and 2 CPGs being No [33, 38]. Of the remaining 3 CPGs, 2 were rated Yes with modifications and Yes [35, 37], while the remaining CPG was rated No and Yes with modifications [32].

### Scaled domain percentage quality assessment (Table 4)

With regards to scaled domain percentages of the overall CPGs, scope and purpose scores ranged from 83.3 to 100.0%, stakeholder involvement scores ranged from 38.9 to 88.9%, rigor-of-development scores ranged from 24.0 to 91.7%, clarity of presentation scores ranged from 38.9 to 100.0%, applicability scores ranged from 10.4 to 50.0%, and editorial independence scores ranged from 37.5 to 95.8%. With regards to scaled domain percentages of the CAM sections, scope and purpose scores ranged from 72.2 to 100.0%, stakeholder involvement scores ranged from 22.2 to 61.1%, rigor-of-development scores ranged from 25.0 to 73.9%, clarity-of-presentation scores ranged from 44.4 to 86.1%, applicability scores ranged from 10.4 to 37.5%, and editorial independence scores ranged from 16.7 to 70.8%.

**Table 4:** Scaled domain percentages for appraisers of each guideline

Guideline		Domain score (%)					
		Scope and purpose	Stakeholder involvement	Rigour of development	Clarity of presentation	Applicability	Editorial Independence
Lam 2019 [32]	Overall Guideline	94.4	55.6	51.1	66.7	10.4	58.3
	CAM Section	94.4	61.1	51.1	66.7	10.4	50.0
Silvestri 2019 [33]	Overall Guideline	83.3	38.9	45.8	38.9	25	66.7
	CAM Section	72.2	22.2	40.6	55.6	12.5	58.3
Riemann 2017 [35]	Overall Guideline	100.0	72.2	58.3	94.4	25.0	70.8
	CAM Section	100.0	55.6	55.2	86.1	22.9	70.8
Qaseem 2016 [37]	Overall Guideline	100.0	88.9	91.7	100.0	25.0	91.7
	CAM Section	94.4	50.0	73.9	66.7	25.0	66.7
Denlinger 2014 [38]	Overall Guideline	83.3	63.9	24.0	47.2	20.8	37.5
	CAM Section	83.3	33.3	25.0	44.4	16.7	16.7
Howell 2013 [39]	Overall Guideline	100.0	66.7	86.5	91.7	50.0	95.8
	CAM Section	91.7	47.2	66.7	83.3	37.5	54.2

### Scope and purpose

The overall objectives and health questions were generally well-defined for both the overall CPGs and the CAM sections of the CPGs. All CPGs scored highly in providing the overall goals of the CPGs and for defining the target disease/condition in the overall CPG and CAM sections. For the population to which the CPGs were meant to apply, all CPGs scored highly for both the overall CPGs and CAM sections [32, 33, 35, 37–39].

### Stakeholder involvement

In terms of the overall CPGs, most provided thorough details regarding the characteristics of the members of the guideline development group, including their names, professions, and institutional affiliation [32, 35, 37–39], while 1 did not [33]. However, for the CAM sections of the CPGs, only 2 CPGs included CAM experts in their guideline development group [32, 39]. In terms of the views and preferences of the target population, only 2 CPGs for both the overall and CAM sections detailed these views [32, 37], while the rest of the CPGs did not [33, 35, 38, 39]. For the overall CPGs, target users were typically well-defined and descriptions included items such as the type or specialty of practitioner [32, 33, 35, 37–39]. For the CAM sections, 3 CPGs defined their target users well [32, 35, 37], while the other 3 did not [33, 38, 39].

### Rigor of development

With the exception of 1 CPG [38], systematic methods were used to search for evidence for the overall CPGs and the CAM sections of the CPGs [32, 33, 35, 37, 39]. For the overall CPGs, the criteria for selecting the evidence were clearly described for 4 CPGs [33, 35, 37, 38], while 2 CPGs did not contain clearly defined criteria for selecting the evidence [32, 38]. For the CAM sections of the CPGs, a few CPGs clearly defined their criteria for selecting evidence [33, 35, 37, 39], and a subset of the CPGs did not [32, 38]. For both the overall CPGs and CAM sections, the strengths and limitations of the body of evidence were clearly described in 4 CPGs [33, 35, 37, 39], while 2 of the CPGs did not identify strengths and limitations well for both the overall and CAM sections [32, 38]. Discrepancies existed between how the methods for formulating recommendations were outlined. For overall CPGs, some provided a sufficient amount of detail on how consensus was reached [35, 37, 39], while others provided little to no information [32, 33, 38]. This was similar for the CAM sections, as some CPGs adhered to the criteria outlined in the AGREE II instrument [35, 37, 39], while others did not [32, 33, 38]. Although quality varied between CPGs, all CPGs considered some of the health benefits, side effects, and/or risks in formulating their recommendations, for both the overall CPGs and CAM sections. All CPGs had an explicit link between their supporting evidence and the recommendations for both the overall and CAM sections. In terms of external review prior to publication, only 2 CPGs mentioned that they were externally reviewed [37, 39], while the rest of them were not [32, 33, 35, 38]. None of the CPGs were externally reviewed by CAM experts prior to publication. Half of the CPGs included a procedure for updating the CPGs [32, 37, 39], while the other half did not include this procedure [33, 35, 38], with respect to both the overall CPGs and CAM sections.

### Clarity of presentation

Most CPGs offered specific and unambiguous recommendations for both the overall CPG and the CAM sections except for 1 CPG [38]. However, many of the CPGs did not include details in their recommendations, such as the identification of the intent/purpose, relevant population, or caveats. In terms of presenting different options for the management of the condition or health issue, 5 CPGs scored highly for both the overall CPGs and the CAM sections, with the exception of 1 CPG which scored poorly [22]. While key recommendations were generally easily identifiable for the overall recommendations in the CPGs [32, 35, 37–39], some CPGs did not have easily accessible key recommendations [33]. CAM recommendations were often more difficult to identify, and a few CPGs did not have easily identifiable key recommendations [32, 33, 37].

### Applicability

One CPG discussed facilitators and barriers to the implementation of the recommendations for the overall CPG and CAM sections [39], while the other 5 CPGs did not for either the overall CPGs or the CAM sections [32, 33, 35, 37, 38]. In terms of providing advice and/or tools to support implementation of the recommendations, 1 CPG did this for both the overall CPG and the CAM section [39], while the rest of the CPGs did not [32, 33, 35, 37, 38]. One CPG addressed the resource implications of implementing the recommendations for both the overall CPG and CAM sections [39], while the other 5 CPGs contained little to no information [32, 33, 35, 37, 38]. No CPGs provided monitoring and auditing criteria for the overall CPGs or the CAM sections.

### Editorial Independence

The 6 CPGs differed in their reporting of the funding source or competing interests of the members of the guideline development panel. Four out of the 6 CPGs stated that the views of the funding body did not influence the content of the CPG [33, 35, 37, 39], while 2 of the 6 CPGs did not state this explicitly [32, 38] for either the overall CPGs or the CAM sections. For the last question in this AGREE II instrument domain, 4 CPGs recorded and addressed the competing interests of the guideline development group [32, 35, 37, 39], while 2 did not address competing interests [33, 38] for neither the overall CPGs nor the CAM sections.

## Discussion

The purpose of this study was to identify the quantity and assess the quality of CAM recommendations in CPGs for the treatment and/or management of insomnia to identify credible, knowledge-based resources for both patients and healthcare professionals to base therapy decisions upon. This study identified 9 CPGs published between 2009 and 2020 that were relevant to the treatment and/or management of insomnia. Out of those 9 CPGs, 6 CPGs made CAM therapy recommendations, 1 CPG mentioned CAM but did not provide CAM therapy recommendations, and 2 CPGs contained neither CAM mentions nor CAM recommendations. The quality of each CPG when assessed by the 23-item AGREE II instrument differed greatly both between the 6 overall CPGs and within the 6 specific domains for each CPG. In assessing the overall CPGs, 2 CPGs scored 5.0 or higher in both average appraisal score and average overall assessment [37, 39], and 4 CPGs scored 4.0 or lower in both of these metrics [32, 33, 35, 38]. In assessing the CAM sections of each CPG, 3 CPGs scored 4.5 or higher in both average appraisal score and average overall assessment [35, 37, 39], and 3 CPGs scored 4.5 or lower in both of these metrics [32, 33, 38] (1 = strongly disagree; 7 = strongly agree that criteria are met).

Of the 6 CPGs making CAM recommendations, yoga was recommended for the treatment and/or management of insomnia in 2 CPGs [38, 39]. These 2 CPGs focused on co-morbid insomnia with cancer as a comorbidity. Herbal remedies were also recommended for the treatment of insomnia in 2 CPGs [32, 33]. One of the CPGs provided recommendations in the context of co-morbid insomnia [32], while the other focused on the treatment of primary insomnia [33]. One guideline recommended against the use of acupuncture, chiropractic techniques, herbal medicines, homeopathy, yoga, mindfulness-based stress relaxation, and foot reflexology [35]. This recommendation against the use of CAM for insomnia may be due to the fact that low-quality evidence was consulted. In future updates of such guidelines, more recent and high-quality evidence should be consulted for the recommendation of CAM for insomnia. The remaining recommendations in the other 6 guidelines were either conflicting or unclear regarding the use of CAM therapies, or no recommendations were provided for the use of CAM therapies for insomnia altogether [32, 33, 35, 37–39]. In order to propose clearer recommendations, the authors of these CPGs should consult more recent and high-quality sources of evidence to inform their recommendations in future updates.

To our knowledge, no previous studies have determined the quantity and quality of CAM therapy recommendations in insomnia CPGs; this is the first study to assess the credibility and nature of such recommendations. In this study, the scaled domain percentages for the overall CPGs from highest to lowest were: scope and purpose (93.5%), clarity of presentation (73.2%), editorial independence (70.14%), stakeholder involvement (64.4%), rigour of development (59.5%), and applicability (26.0%). The scaled domain percentages for the CAM sections of the CPGs from highest to lowest were: scope and purpose (89.4%), clarity of presentation (67.1%), editorial independence (52.8%), rigour of development (52.1%), stakeholder involvement (44.9%), and applicability (20.8%). These findings are similar to the results of guideline assessments for  other conditions/diseases in the context of CAM recommendations. Various other studies which used a similar format to this current study found similar results in terms of scaled domain percentages for overall CPGs and CAM sections. For example, 1 study reported that across 17 low back pain  CPGs, scaled domain percentages from highest to lowest were as follows: scope and purpose, clarity of presentation, stakeholder involvement, rigor of development, editorial independence, and applicability [41]. Other studies assessing the quality of CAM recommendations across arthritis, lung cancer, hypertension, depression, and cancer-related pain CPGs also found similar trends to exist whereby the order of domains were similar with the scope and purpose and clarity of presentation domains typically scoring higher, and the domain of applicability typically scoring lower [42–46]. These findings reflect the fact that the variable and sometimes sub-optimal quality of CPGs is not a unique phenomenon.

Overall, this study revealed that very few CPGs containing CAM recommendations exist to support evidence-informed decision making among patients and their healthcare providers for the treatment and/or management of insomnia. Apart from a lack of evidence surrounding many CAM therapies, this lack of CAM recommendations for insomnia found in CPGs could also be explained by other factors which can impact the availability of CAM research, including the following: negative attitudes about CAM therapies [47–51], and a lack of CAM funding [52–55]. Despite this, CAM is utilized by more than 40% of the population in some parts of the world [56, 57]; for example, 36% of adults use some form of CAM in the USA [58]. Thus, this should serve as a reminder to researchers and clinicians alike that the importance of CAM research and recommendation implementation in CPGs comprises a large component of patient choice. It is therefore important for future CPG developers to utilize the available resources to optimize CPGs. The AGREE II instrument, along with various other principles, frameworks, criteria and checklists are available to assist CPG developers, including CAM CPG developers, to generate the highest-quality CPGs [59–64].

### Strengths and limitations

One notable strength of this study included a comprehensive systematic review methodology to identify eligible CPGs which focused on insomnia management and/or treatment. Another strength included the use of the AGREE II instrument to assess CPGs, as it has been found to be both reliable and valid, and is the internationally-accepted gold standard for appraising CPGs. A possible limitation to the interpretation of our findings includes the fact that the CPGs were independently assessed by 2 assessors, instead of 4 as recommended by the AGREE II instrument instruction manual. To reduce discrepancies and standardize scoring, a pilot-test was conducted by JYN, NDP, and an another research assistant where 3 CPGs were independently appraised, scores were discussed, and consensus was achieved on how to apply the AGREE II instrument. After the 6 eligible CPGs were assessed, JYN met with NDP and the another research assistant to discuss and resolve uncertainties. Lastly, it should be acknowledged that we excluded non-English language CPGs, however, many traditional systems of medicine originate from regions of the world where English is not commonly spoken (i.e. traditional Asian medicine in China, Japan and Korea, among others).

## Conclusions

This study identified 6 eligible CPGs published between 2009 and 2020 which provided CAM recommendations for insomnia, inclusive of acupuncture, yoga, herbal medicines, and mind-body practices. Following the appraisal of these CPGs with the AGREE II instrument, it was found that the quality varied within and across these CPGs. The CPGs which received higher scores could be utilized to inform healthcare providers about specific CAM therapies and could serve as the foundations for discussions involving the use of evidence-based CAMs for treating/managing insomnia. In future updates of CPGs, those which achieved lower domain scores and overall recommendations could be improved according to specifics in the AGREE II instrument, among other high-quality guideline development and implementation resources. At present, the lack of high-quality CAM recommendations across this subset of CPGs may lead to the continued use of potentially harmful CAM therapies, or the underuse of beneficial CAM therapies. These findings are important in justifying the need to understand why more CAM therapies have yet to be incorporated in insomnia CPGs despite great patient preference, and to determine where gaps in current knowledge exist. Future research should also seek to identify and incorporate into CPGs, CAM therapies which were not necessarily reviewed here, but which are supported by a sufficient evidence-base. Ultimately, this study suggests that while many patients utilize CAM for the treatment/management of insomnia, evidence-based recommendations are limited across CPGs, and further research is warranted to assist healthcare providers in standardizing the use of CAM therapies as part of patient care. 

## Supplementary Information

**Additional file 1: Supplementary File 1.** MEDLINE Search Strategy for Insomnia Clinical Practice Guidelines Executed April 17, 2020.

**Additional file 2: Supplementary File 2.** Modified AGREE II Questions Used to Guide Scoring of CAM Sections of Each Guideline.

## Data Availability

All relevant data are included in this manuscript.

## Abbreviations

AGREE II: Appraisal of Guidelines for Research & Evaluation II
CAM: Complementary and alternative medicine
CPG: Clinical practice guideline
ECRI: Emergency Care Research Institute
NCCIH: National Center for Complementary and Integrative Health
NICE: National Institute for Health and Care Excellence
PICO: Population, Intervention, Comparison and Outcomes
PRISMA: Preferred Reporting Items for Systematic Reviews and Meta-Analyses
