# Supplementary material for: A systematic review and quality assessment of complementary and alternative medicine recommendations in insomnia clinical practice guidelines
Source: BMC Complement Med Ther. 2021 Feb 8;21:54. doi: 10.1186/s12906-021-03223-3 (PMC7869239; doi:10.1186/s12906-021-03223-3)
Supplement: Supplementary file 1 — Additional file 1: Supplementary File 1. MEDLINE Search Strategy for Insomnia Clinical Practice Guidelines Executed April 17, 2020. [file 12906_2021_3223_MOESM1_ESM.docx]

## Supplementary File 1: MEDLINE Search Strategy for Insomnia Clinical Practice Guidelines Executed Apr 17, 2020

| Database: Ovid MEDLINE(R) and Epub Ahead of Print, In-Process & Other Non-Indexed Citations, Daily and Versions(R) <1946 to April 16, 2020>  Search Strategy:  --------------------------------------------------------------------------------  1 insomnia.mp. or "Sleep Initiation and Maintenance Disorders"/ (25067)  2 limit 1 to ("all infant (birth to 23 months)" or "all child (0 to 18 years)" or "newborn infant (birth to 1 month)" or "infant (1 to 23 months)" or "preschool child (2 to 5 years)" or "child (6 to 12 years)" or "adolescent (13 to 18 years)") (3926)  3 1 not 2 (21141)  4 limit 3 to (english language and humans and yr="2009 -2020" and (guideline or practice guideline)) (19)  *************************** |
| --- |

## 
